# Neuroglucopenia and Metabolic Distress in Two Patients with Viral Meningoencephalitis: A Microdialysis Study

**DOI:** 10.1007/s12028-016-0272-8

**Published:** 2016-04-25

**Authors:** Mario Kofler, Alois Schiefecker, Ronny Beer, Florian Sohm, Gregor Broessner, Paul Rhomberg, Peter Lackner, Bettina Pfausler, Claudius Thomé, Erich Schmutzhard, Raimund Helbok

**Affiliations:** 1 Neurological Intensive Care Unit, Department of Neurology, Medical University of Innsbruck, Anichstrasse 35, 6020 Innsbruck, Austria; 2 Department of Neurosurgery, Medical University of Innsbruck, Anichstrasse 35, 6020 Innsbruck, Austria; 3 Department of Radiology, Medical University of Innsbruck, Anichstrasse 35, 6020 Innsbruck, Austria

**Keywords:** Cerebral microdialysis, Encephalitis, Multimodal neuromonitoring, Brain tissue glucose, Neuroglucopenia

## Abstract

**Introduction:**

Viral encephalitis is an emerging disease requiring intensive care management in severe cases. Underlying pathophysiologic mechanisms are incompletely understood and may be elucidated using invasive multimodal neuromonitoring techniques in humans.

**Methods:**

Two otherwise healthy patients were admitted to our neurological intensive care unit with altered level of consciousness necessitating mechanical ventilation. Brain imaging and laboratory workup suggested viral encephalitis in both patients. Invasive neuromonitoring was initiated when head computed tomography revealed generalized brain edema, including monitoring of intracranial pressure, brain metabolism (cerebral microdialysis; CMD), brain tissue oxygen tension (in one patient), and cerebral blood flow (in one patient).

**Results:**

Brain metabolism revealed episodes of severe neuroglucopenia (brain glucose <0.7 mM/l) in both patients, which were not attributable to decreased cerebral perfusion or hypoglycemia. CMD-glucose levels changed depending on variations in insulin therapy, nutrition, and systemic glucose administration. The metabolic profile, moreover, showed a pattern of non-ischemic metabolic distress suggestive for mitochondrial dysfunction. Both patients had a prolonged but favorable clinical course and improved to a modified Rankin Scale Score of 1 and 0 three months later.

**Conclusion:**

Invasive multimodal neuromonitoring is feasible in poor-grade patients with viral meningoencephalitis and may help understand pathophysiologic mechanisms associated with secondary brain injury. The detection of neuroglucopenia and mitochondrial dysfunction may serve as treatment targets in the future.

## Introduction

Encephalitis is defined as inflammation of the brain parenchyma associated with neurologic dysfunction [1]. Clinical symptoms are varied and etiology often remains unknown despite a broad diagnostic workup, having viruses as most common etiologic agents [2]. Ten percent to 25 % of patients deteriorate into a comatose state necessitating mechanical ventilation [2].

Management of poor-grade patients with viral encephalitis requiring intensive care medicine focuses on antiviral therapy, if available [3], and the treatment of complications including seizures and elevated intracranial pressure (ICP) [2, 4, 5]. ICP monitoring is a useful adjunct and may be considered in patients with severe brain edema [6]. Newer technologies allow online monitoring of local cerebral perfusion (cerebral blood flow, CBF), brain tissue oxygen tension (P_bt_O_2_), and brain metabolism (cerebral microdialysis, CMD). The feasibility of these additional bedside monitoring techniques has been demonstrated in patients with meningitis [7, 8].

Investigating the brain metabolic profile in patients with viral encephalitis seems crucial to better understand pathophysiologic mechanisms leading to secondary brain injury, which are so far only poorly understood. Viruses lack the ability of autonomous energy production and therefore need to take control over their host cells’ metabolism in order to replicate. Cell culture experiments indicate that glucose uptake is increased and glucose utilization by glycolysis and the pentose phosphate way is enhanced during viral infection, which leads to lower brain extracellular glucose concentrations [9, 10]. Low CMD-glucose levels are associated with poor functional outcome in patients with traumatic brain injury (TBI) and subarachnoid hemorrhage (SAH) and occur in bacterial meningitis [7, 11, 12]. Furthermore, several changes of enzyme activity in the tricarboxylic acid (TCA) cycle and the electron transport chain (ETC), thus an alteration of mitochondrial function, have been described in cultured cells [10, 13]. Recently, a pattern suggestive for mitochondrial dysfunction has been described using brain metabolic information derived from CMD, defined as elevated lactate or lactate-to-pyruvate-ratio (LPR), with normal to increased pyruvate (while low CMD-pyruvate may be an accompaniment of ischemia) [14], more specifically as LPR >30 and CMD-pyruvate >70 µM/l [15].

Here we describe the brain metabolic profile and the clinical course in two patients with severe viral meningoencephalitis. Both patients gave written informed consent for the publication of these data.

## Case One

**Fig. 1:**
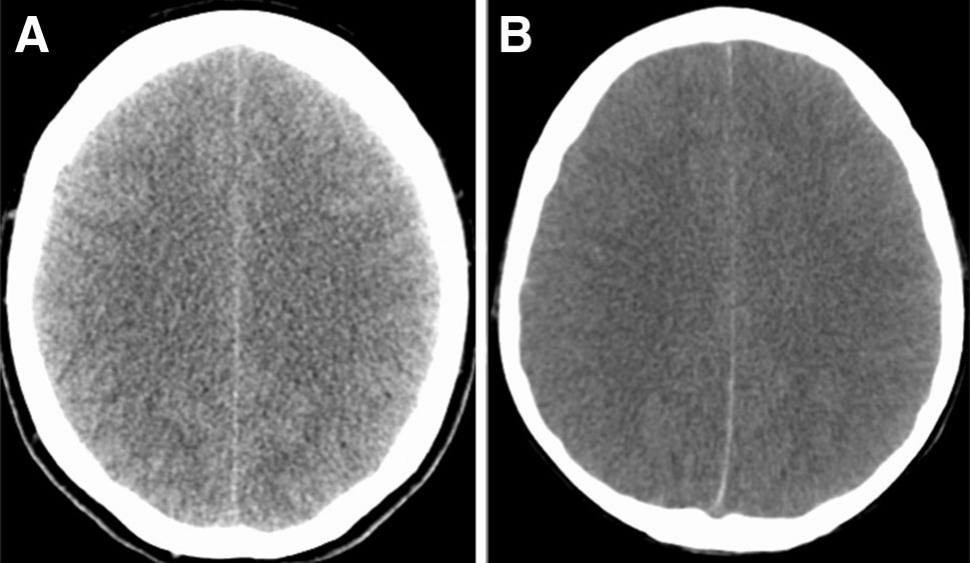
Axial computed tomography (CT) scans on admission demonstrating global cerebral edema in patient one (**a**) and two (**b**)

A 38-year-old, otherwise healthy, woman presented to an external hospital with drowsiness and aphasia after a week of recurrent episodes of fever (>39 °C) accompanied by muscle weakness and an exanthema of trunk, arms, and legs. Travel- and exposure-history was uneventful and routine vaccination was performed. Initial computed tomography (CT) of the head was normal, and cerebrospinal fluid (CSF) analysis revealed a mild pleocytosis (3 lymphocytes and 1 granulocyte per µl), elevated protein of 311 mg/dl (normal range 15–40 mg/dl), and a normal glucose concentration (>60 % of serum glucose). Neurologic examination revealed a patient with altered level of consciousness, mixed aphasia, and neck stiffness. Serology for Tick-borne encephalitis virus (TBEV) and *Borrelia burgdorferi*, as well as polymerase chain reaction (PCR) of herpes simplex virus (HSV), varicella-zoster virus (VZV), *Enterovirus,* and *Mycobacterium tuberculosis* remained negative in serum and CSF samples. Serum C-reactive protein (CRP) was 4.21 mg/dl (normal range <0.85 mg/dl). Neuroimaging showed meningeal enhancement, supporting the clinically and laboratory suspected diagnosis of meningoencephalitis. Intravenous acyclovir (750 mg TID), ceftriaxone (2 g BID), and moxifloxacin (400 mg SID) were started. Twelve hours later, the patient developed repeated seizures, necessitating mechanical ventilation. Follow-up CT of the brain showed diffuse brain swelling (Fig. 1a), and she was transferred to our unit. Brain magnetic resonance imaging (MRI) revealed signal enhancement of the caput nuclei caudati as well as bilateral hyperintense white matter changes of the perivascular space in fluid-attenuated inversion recovery (FLAIR) sequence, suggestive for viral encephalitis. West-Nile virus (WNV) IgM-antibodies were detected in serum specimens. HIV, Hepatitis B and C virus, *Borrelia burgdorferi*, *Listeria monocytogenes*, TBEV, Hantavirus, Chikungunya virus, and Sandfly virus serology were all negative, so was PCR screening for HSV, VZV, Human cytomegalovirus (CMV), Epstein-Barr virus (EBV), Human herpesvirus 6 (HHV6) and enterovirus. Due to the patient’s clinical and radiologic deterioration, multimodal neuromonitoring probes were inserted for the assessment of ICP (NEUROVENT-P-TEMP, Raumedic^®^, Helmbrechts, Germany), P_bt_O_2_ (Licox^®^, Integra LifeSciences, Saint Priest, France), and brain metabolism (71 High Cut-Off Brain Microdialysis Catheter, M Dialysis AB, Stockholm, Sweden). Probe location in normal-appearing white matter was confirmed by CT of the brain.

**Fig. 2:**
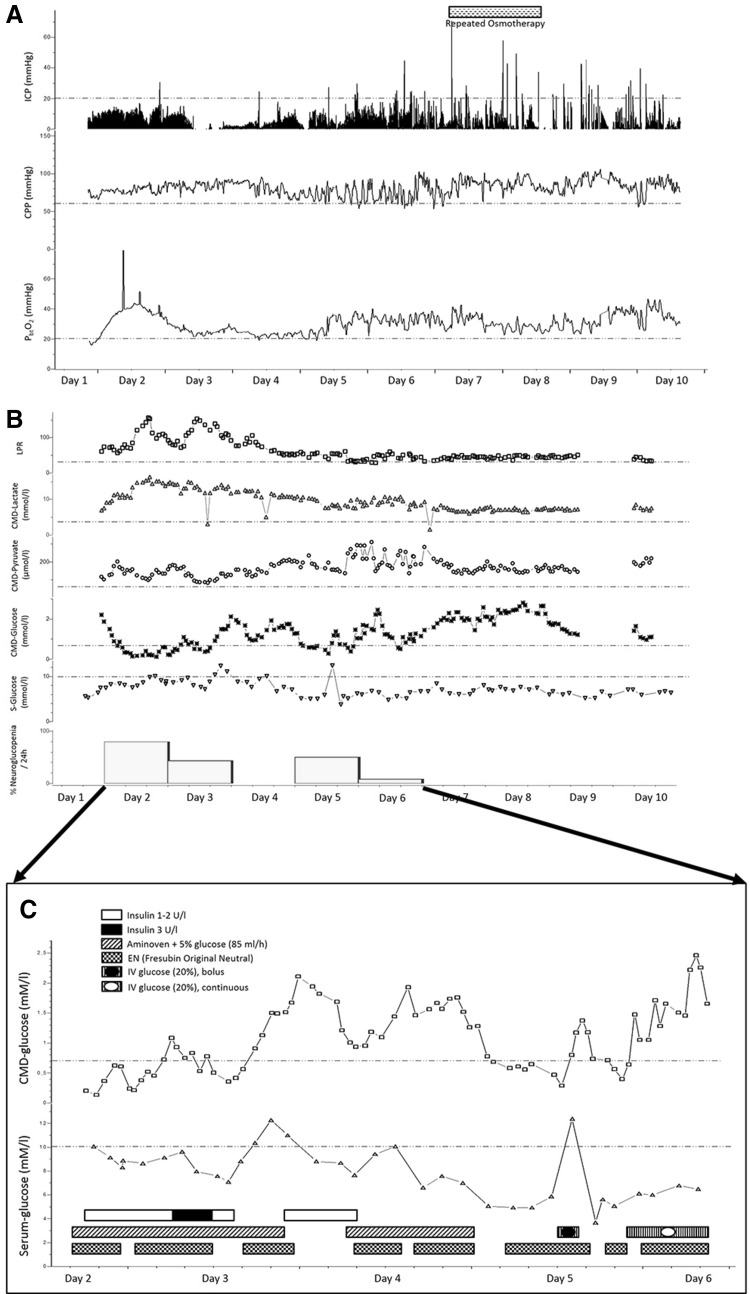
Monitoring and metabolic profile of patient one. **a** Continuous values of intracranial pressure (ICP) and cerebral hemodynamic parameters [cerebral perfusion pressure (CPP), brain tissue oxygen tension (P_bt_O_2_)] over the neuromonitoring period; *horizontal bar* represents the period of repeated osmotherapy, **b** hourly values of brain metabolism assessed by cerebral microdialysis (CMD) and levels of serum (S-)-glucose over the neuromonitoring period; *vertical bars* represent the percentage of episodes of neuroglucopenia (CMD-glucose <0.7 mM/l) per 24 h; *LPR* lactate-to-pyruvate-ratio, **c** Changes in brain and serum glucose levels associated with nutrition and systemic glucose management [continuous intravenous insulin, enteral nutrition (EN), amino acid supplementation, and intravenous (IV) glucose, shown as *horizontal bars*] from day two to six. *Dashed lines* indicate published thresholds for normal values of the respective parameters: ICP <20 mmHg, CPP >60 mmHg, P_bt_O_2_ >20 mmHg, LPR <30, CMD-lactate <4 mmol/l, CMD-pyruvate >70 µmol/l, CMD-glucose >0.7 mmol/l, serum glucose <10 mmol/l

During the observation time, ICP remained <20 mmHg in 99 % of monitoring time, P_bt_O_2_ dropped to 16 mmHg during the initial hours and increased to normal levels after immediate augmentation of cerebral perfusion pressure (CPP) to >70 mmHg. All values remained stable during the neuromonitoring time, except for fluctuations of CPP without decrease of P_bt_O_2_ during days five and six (Fig. 2a). Parenteral amino acid supplementation (Aminoven^®^ 3.5 %, Fresenius Kabi Austria, Graz, Austria) was started at 10 h, enteral nutrition (EN) (Fresubin^®^ Original Neutral, Fresenius Kabi Austria) at 27 h after admission. Intravenous amino acid infusions are given until 70 % of the calculated energy demand are reached with EN. Intravenous insulin was started shortly after EN at a rate of 2 U/h when serum glucose concentration reached 10 mM/l (180 mg/dl). Daily median brain interstitial glucose concentration ranged from 0.35 mM/l (IQR 0.2–0.65, day two) to 2.38 mM/l (IQR 2.2–2.5, day eight). In 23 % of monitoring time CMD-glucose was <0.7 mM/l, in 3 % of time <0.2 mM/l. Our standard serum glucose protocol aims at maintaining systemic glucose levels between 6.1 and 8.3 mM/l (110–150 mg/dl). When neuroglucopenia was detected, we initiated a more liberal glucose regimen, allowing values up to 10 mM/l (180 mg/dl).

**Fig. 3:**
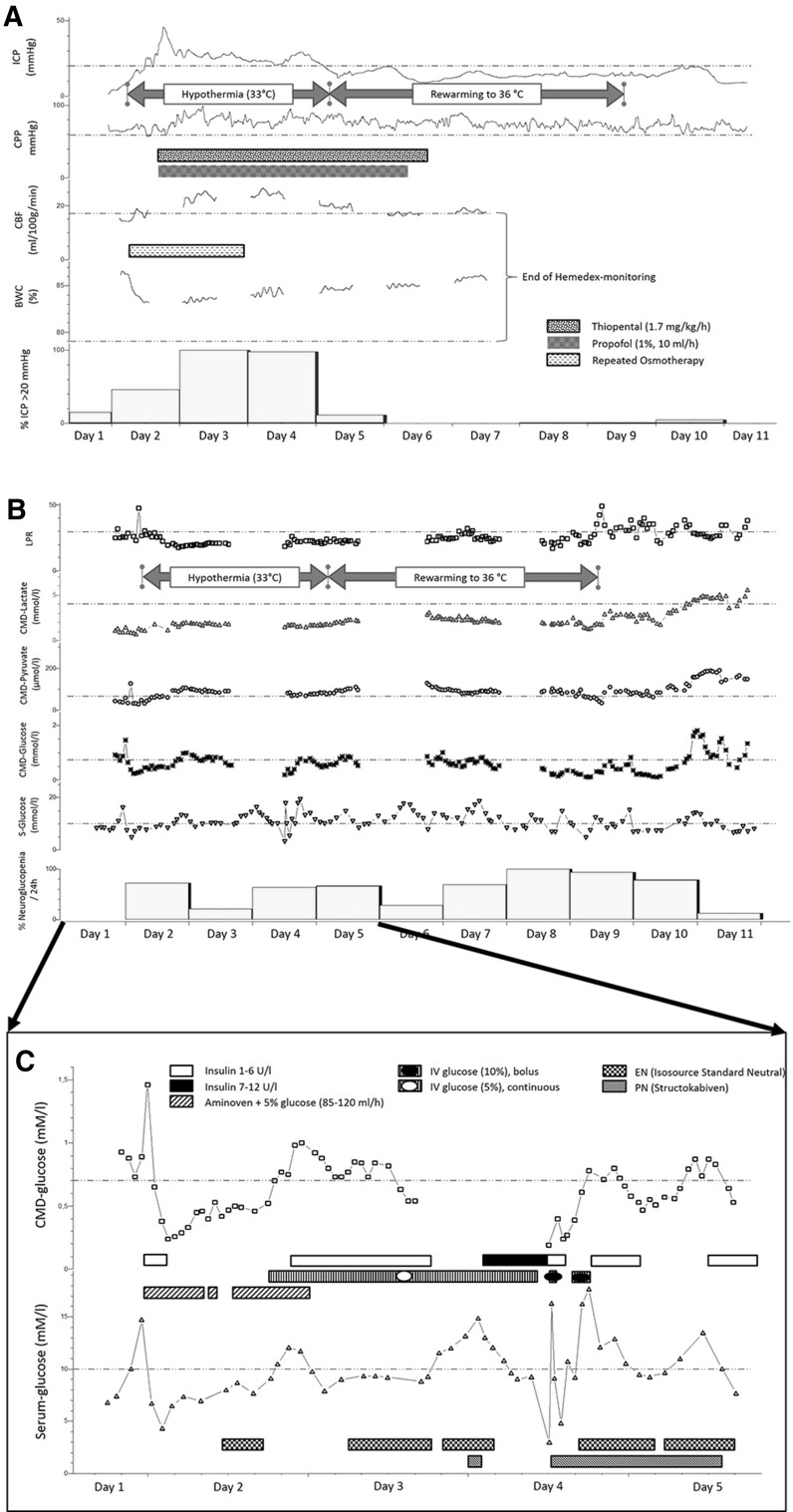
Monitoring and metabolic profile of patient two. **a** Continuous values of intracranial pressure (ICP) and cerebral hemodynamic parameters [cerebral perfusion pressure (CPP), cerebral blood flow (CBF), brain water content (BWC)] over the neuromonitoring period; *vertical bars* represent the percentage of elevated ICP (>20 mmHg) per 24 h; *horizontal bars* represent the periods of repeated osmotherapy, thiopental, and propofol administration; *arrows* represent the duration of targeted temperature management (33 °C) and slow rewarming to 36 °C, **b** hourly values of brain metabolism assessed by cerebral microdialysis (CMD) and levels of serum (S-)-glucose over the neuromonitoring period; *vertical bars* represent the percentage of neuroglucopenia (CMD-glucose <0.7 mM/l) per 24 h; *arrows* represent the duration of targeted temperature management (33 °C) and slow rewarming to 36 °C; *LPR* lactate-to-pyruvate-ratio. **c** Changes in brain and serum glucose levels associated with nutrition and systemic glucose management [continuous intravenous insulin, enteral nutrition (EN), parenteral nutrition (PN), amino acid supplementation, and intravenous (IV) glucose shown as *horizontal bars*] from day one to five. *Dashed lines* indicate published thresholds for normal values of the respective parameters; ICP <20 mmHg, CPP >60 mmHg, CBF >17 ml/100 g/min, LPR <30, CMD-lactate <4 mmol/l, CMD-pyruvate >70 µmol/l, CMD-glucose >0.7 mmol/l, serum glucose <10 mmol/l

Serum glucose measurements were above 6 mM/l in 79 % of analyses. Cerebral glucose levels were influenced by modifications in insulin therapy, nutrition, and systemic glucose infusion, as detailed in Fig. 3c. Briefly, insulin application or dose augmentation was associated with a decrease in CMD-glucose and episodes of neuroglucopenia in the early phase of neuromonitoring. Stopping insulin infusion due to neuroglucopenia preceded a rapid increase in both systemic and cerebral glucose. On day 4, when the patient received combined EN and parenteral amino acid supplementation, brain glucose levels remained above 0.7 mM/l. When the caloric requirements were reached with EN, Aminoven^®^ was stopped. This was associated with a decrease in cerebral glucose to <0.7 mM/l. Bolus systemic glucose infusion (20 %) was associated with an intermediate increase in systemic and brain glucose levels. Overall, however, we did not find a significant correlation (Spearman’s rho) between serum and CMD-glucose (*p* = 0.38).

CMD-lactate and lactate-to-pyruvate-ratio (CMD-LPR) was constantly elevated (>4 mM/l in 98 % of measurements and >30 mM/l in 99 % of measurements), and CMD-pyruvate was within normal range (>70 µM/l in 99 % of measurements). The brain metabolic profile suggested non-ischemic metabolic distress, confirmed by P_bt_O_2_ levels >20 mmHg, recently defined as mitochondrial dysfunction (Fig. 2b) [15].

From day six on, the patient developed short episodes of raised ICP without significant decrease in P_bt_O_2_, which were successfully treated with hypertonic saline (10 %, 100 ml).

Clinical condition improved and the patient was extubated on day ten. At that time she followed commands, neurologic examination did not reveal any focal motor deficit, but the patient still suffered from encephalopathy with disorientation to time and person. Her neuropsychological status improved over the next weeks, and she could be discharged to an external hospital without neurologic deficit.

## Case Two

A 17-year-old, otherwise healthy, male patient presented with an upper respiratory tract infection and was treated with amoxicillin (dose not available). He was admitted to a peripheral hospital when he developed skin rash and muscle pain. Physical examination revealed tonsillitis and cervical lymphadenopathy; neurological examination was normal at this time. Differential blood count showed monocytosis (18 %, normal range 1–12 %); CRP was elevated (1.21 mg/dl, normal range <0.5 mg/dl).

Within 24 h, the patient developed drowsiness and neurologic exam showed neck stiffness. CSF analysis revealed mild pleocytosis (7 cells/µl, normal range 0–4/µl) and normal glucose levels (CSF/serum glucose ratio of 0.66, normal range >0.6). Antiviral therapy with acyclovir (500 mg TID) and antimicrobial chemotherapy with ceftriaxone (2 g BID) was initiated. The patient further deteriorated and was mechanically ventilated and transferred to our unit. On arrival, neurologic exam revealed bilateral non-reactive dilated pupils, which most likely developed during the 30 min of helicopter transfer. Head CT scan demonstrated global cerebral edema (Fig. 1b) with transtentorial herniation. The patient immediately underwent bilateral craniectomy. Neuromonitoring probes were placed into the right frontal white matter to measure ICP, brain metabolism, CBF, and brain water content (BWC) (HEMEDEX QFlow500™ Perfusion Probe, HEMEDEX™, Cambridge, MA). A broad diagnostic workup revealed Epstein-Barr virus (EBV)-associated encephalitis confirmed by serology (IgM/IgG antibodies in CSF and serum) and PCR (EBV-DNA in CSF). Intravenous ganciclovir (300 mg BID) and doxycycline (100 mg BID) treatment was initiated.

After craniectomy, ICP was still elevated (episodes of ICP >30 mmHg) and decreased only after osmotherapy, therapeutic hypothermia (33 °C), as well as thiopental (1.7 mg/kg/h) and propofol infusion (1 %, 10 ml/h), aiming to achieve a burst-suppression pattern on surface electroencephalogram (EEG). CBF was low (median 16 ml/100 g/min, IQR 13.7–18.1) during the first 24 h of monitoring and increased thereafter. BWC started at 86 % and decreased to 82 % during repeated osmotherapy concomitant to radiologic edema regression (significantly lower BWC values around 70 % have been described in normal-appearing brain tissue on head CT scans in acutely brain-injured patients) [16]. As ICP normalized on day four, intravenous thiopental and propofol infusions could be gradually reduced; slow rewarming was initiated, and the patient’s body temperature reached 36 °C on day nine of monitoring (Fig. 3a).

During the time of hypothermia, CMD-glucose levels were low (64 % of measurements <0.7 mM/l, 4 % <0.2 mM/l), despite a liberal systemic glucose management. Overall, 80.7 % of systemic glucose measurements were >6 mM/l. Insulin application (3 U/h) was started together with parenteral amino acid supplementation after 11 h, EN (Isosource^®^ Standard Neutral Smartflex^®^, Nestlé Austria, Vienna, Austria) was started after 22 h. CMD-lactate, CMD-pyruvate, and CMD-LPR were within normal range [median of 1.9 mM/l (IQR 1.7–2.2), 84 µM/l (IQR 69.3–93.7) and 23 (IQR 21.4–25.5), respectively].

After the patient’s body temperature reached 36 °C, CMD-glucose levels remained low (54 % <0.7 mM/l, 11 % <0.2 mM/l), CMD-lactate and CMD-LPR exceeded pathologic thresholds for extended periods of time (32.4 % >4 mM/l and 46.3 % >30, respectively). As CMD-pyruvate levels were at a median of 92 µM/l (IQR 71–149) and 94 % >70 µM/l, several episodes (32.7 % of measurements) of mitochondrial dysfunction were observed (Fig. 3b).

When neuroglucopenia was detected, ICP was within normal range (<20 mmHg) in 80.2 %, CPP was >60 mmHg in 97.7 %, and CBF was >17 ml/100 g/min in 69.2 % of time. An association between systemic and CMD-glucose levels is shown in Fig. 3c. Serum- and CMD-glucose levels were significantly correlated (*r* = 0.47, *p* < 0.0001); insulin-dosage was negatively associated with CMD-glucose levels, and intermittent pauses of EN and PN were associated with a decrease in CMD-glucose.

The patient gradually improved and could be extubated after 23 ventilator days. He showed an atactic-ballistic movement disorder, discrete paresis of the lower limb (especially dorsal flexors), and transient psychosis. He could be discharged to an acute rehabilitation facility after 7 weeks. Fortunately, he improved within 3 months to a normal functional level with only mild neuropsychological deficits (modified Rankin Scale Score of 1, respectively).

## Discussion

Here we present two patients with severe viral meningoencephalitis with global cerebral edema necessitating bilateral craniectomy in one patient. In both patients, clinical course was complicated by raised intracranial pressure, which was successfully treated with osmotherapy (Case One) or osmotherapy in combination with deep sedation and hypothermia (Case Two). Cerebral metabolic profile revealed episodes of neuroglucopenia despite a liberal systemic glucose regimen suggesting increased glucose consumption. Moreover, both patients developed metabolic distress in the absence of brain tissue hypoxia, suggestive for mitochondrial dysfunction [14, 15].

Little is known about brain metabolic changes during viral encephalitis in humans. Viruses interact with cellular metabolic pathways in order to generate energy for replication. Experimental data suggest an important role of glucose metabolism, as cellular glucose uptake is increased and glycolytic flux is enhanced during viral infection in a variety of tissues including neuronal cells, leading to low interstitial glucose levels [9, 10]. Diminished energy reserves, signaled by a reduced level of adenosine triphosphate (ATP) and other nucleoside triphosphates, have been found in virus-infected cells, despite an augmented utilization of glucose [9]. The interaction between viruses and glycolysis may be especially important in neuronal tissue because the brain relies almost entirely on glucose for energy production. In neuronal cell cultures, the inability of cells to increase ATP production required for both viral replication and cellular homeostasis, despite increased glucose consumption, was associated with energy collapse and cell death [10].

In our patients, it is likely that neuroglucopenia occurred secondary to increased glucose metabolism as CBF and P_bt_O_2_ were normal. This increased metabolism is probably due to the higher energy demand owing to viral replication [9, 10]. Neuroglucopenia is associated with metabolic distress and poor outcome in patients with subarachnoid hemorrhage and TBI [11, 12]. Glucose deprivation leads to swelling of cortical cells (cytotoxic edema) in the experimental setting and SAH patients with global cerebral edema, which was a complicating factor in our patients, showed a trend towards lower CMD-glucose levels [17, 18].

Several interventions may influence brain extracellular glucose concentrations. CMD-glucose levels largely depend on systemic glucose availability and, therefore, glucose delivery. Enteral feeding is associated with higher systemic glucose levels in SAH patients [19]. In our patients, CMD-glucose levels decreased when EN or PN were intermittently stopped. The effect of insulin on systemic glucose utilization was found to be greater than on cerebral glucose metabolism [20]. In our patients, insulin administration was associated with both, a decrease in systemic and brain glucose levels. The relative decrease of CMD-glucose levels following insulin infusion was more pronounced than the drop of systemic glucose levels. This may be in line with a finding in SAH patients in which, under conditions of brain metabolic distress, insulin treatment decreased brain glucose levels independent of systemic glucose concentration [19]. However, the reduction of serum glucose may still have aggravated the decrease in brain glucose. The administration of IV glucose infusion (low volume) recently discussed by experts, however, still needs confirmatory evidence [21]. Such an intervention should carefully take into account the CMD-catheter location (preferably in normal-appearing brain tissue on head-CT), systemic glucose concentration (prevent hyperglycemia), CPP, CBF, and other brain metabolic parameters including the LPR. Neuroglucopenia may also occur when glucose delivery does not meet the increased metabolic demand, which may occur in several conditions including fever, seizures [22], and global cerebral edema (GCE) [18]. The normalization of brain glucose levels may compensate the increased demand due to viral replication and provide energy needed for cellular homeostasis.

In our patients, the brain metabolic profile suggested mitochondrial dysfunction. Beyond glycolysis, viruses interact with the expression of enzymes involved in the TCA cycle and the complexes of the ETC for energy production in order to replicate [10, 13]. Mitochondria are further involved in immune responses and apoptosis control, which may also be altered by viral infection [13]. The diagnosis of mitochondrial dysfunction in humans remains challenging. Positron emission tomography (PET) in combination with CMD has shown that metabolic distress (LPR >40) may occur without ischemia and brain tissue hypoxia in TBI patients [23]. Recently, a metabolic pattern derived from CMD suggestive for mitochondrial dysfunction has been described in SAH and meningitis [7, 15]. However, this approach, involving elevated LPR >30, needs further investigation. It also does not provide information concerning the point of interaction between viral infection and mitochondrial dysfunction. Mitochondrial dysfunction has been discussed as a factor in the emergence of GCE [18], but so far its impact on clinical course and functional outcome has not been clarified [7, 15]. Moreover, nonconvulsive electrographic seizures are associated with metabolic distress in TBI patients [22]. We did not perform continuous EEG monitoring during the entire clinical course in our patients and may have missed subclinical seizures as etiology of cerebral metabolic distress and mitochondrial dysfunction. In Patient 2, continuous EEG monitoring was applied during barbiturate coma aiming to achieve a burst-suppression pattern. The cerebral metabolic pattern did not suggest mitochondrial dysfunction during that time.

Although currently no specific treatment addressing mitochondrial dysfunction exists, animal data suggest a potential effect of cyclosporine A, malibatol A, and inhibitors of the interaction between postsynaptic density protein 95 and the neuronal nitric oxide synthase [24–26].

A case report of a patient with meningoencephalitis reported a cerebral LPR within normal range [8]. Unfortunately, glucose values were not described and head CT scan suggests microdialysis catheter positioning adjacent to the cortex, which may explain differences.

In our patients, we did not observe a temporal relation between neuroglucopenia and mitochondrial dysfunction, suggesting that both phenomena may occur independently based on distinct pathophysiological mechanisms.

The interpretation of our results has several limitations. First, findings are associations; and a causative relationship between viral infection and neuroglucopenia or mitochondrial dysfunction cannot be concluded. Cerebral microdialysis depicts only a small area of the brain, and we cannot conclude that our findings represent the whole brain parenchyma. We also did not perform simultaneous PET scans, which would facilitate an extrapolation of the described metabolic pattern to areas not depicted by cerebral microdialysis. Nevertheless, CMD-catheters were well placed in normal-appearing brain tissue (confirmed by neuroimaging) and therefore may be representative for a larger area in the brain. Further, cell culture and animal data support these findings; and it is very unlikely that interventions secondary to cerebral edema and elevated ICP may have substantially influenced the associations found. Both patients received osmotherapy. In stroke patients, mannitol reduces ICP and LPR but does not affect cerebral glucose [27]. In our patients, hypertonic saline or mannitol did not have a considerable effect on cerebral metabolism, but ICP elevations were effectively controlled in Patient 1 and BWC decreased in Patient 2. It is very unlikely that our sedation protocol provoked episodes of low CMD-glucose levels. The effect of propofol and barbiturates remains controversial; however, metabolic suppression is commonly associated with increasing or stable brain glucose levels [19, 28, 29]. In a microdialysis study on severe head injury, patients receiving pentobarbital had lower CMD-glucose levels and lower LPR [30]. However, the small sample size (2 of 20 patients) and the diversity of underlying pathologies (SAH, ICH, TBI, and cerebral infarction) may lower the significance of these findings. We did not observe a change in CMD-glucose when thiopental or propofol was initiated or stopped.

According to experimental evidence, hypothermia leads to a reduction in cerebral energy demand, a shift towards aerobic glycolysis, and, specifically, lower LPR [31]. In our patient, hypothermia seemed to have the largest impact on cerebral metabolism, as the LPR slightly increased over the rewarming period and markedly increased when the patient’s body temperature reached 36 °C. Lactate levels did not exceed the pathologic threshold of 4 mM/l, indicating enhanced anaerobic glycolysis, during the time of hypothermia. An effect on glucose levels, however, was not observed. As episodes of metabolic distress mainly occurred after rewarming, therapeutic hypothermia may be considered as potential future treatment of mitochondrial dysfunction.

## Conclusion

Invasive multimodal neuromonitoring is feasible in patients with viral encephalitis necessitating mechanical ventilation. Neuroglucopenia occurred frequently in both patients and was not attributable to restricted cerebral perfusion or hypoglycemia. Changes in brain glucose levels were closely associated with changes in systemic glucose levels. Our data suggest that maintaining more liberal values of serum glucose and cautious use of insulin may reduce the occurrence of neuroglucopenia. Brain cellular mitochondrial dysfunction may also contribute to secondary brain injury in patients with viral encephalitis and may be modifiable by therapeutic hypothermia.
